# Simply Scan—Optical Methods for Elemental Carbon Measurement in Diesel Exhaust Particulate

**DOI:** 10.1093/annhyg/meu037

**Published:** 2014-06-17

**Authors:** James A. Forder

**Affiliations:** Analytical Sciences Unit, Health Safety Laboratory, Harpur Hill Industrial Estate, Buxton, Derbyshire SK17 9JN, UK

**Keywords:** BC, black carbon, DEP, diesel exhaust, EC, elemental carbon, exposure assessment, non-destructive, optical method

## Abstract

This article describes a performance assessment of three optical methods, a Magee Scientific OT21 Transmissometer, a Hach-Lange Microcolor II difference gloss meter, and a combination of an office scanner with Adobe Photoshop software. The optical methods measure filter staining as a proxy for elemental carbon in diesel exhaust particulate (DEP) exposure assessment and the suitability of each as a replacement for the existing Bosch meter optical method. Filters loaded with DEP were produced from air in a non-coal mine and the exhaust gases from a mobile crane. These were measured with each apparatus and then by combustion to obtain a reference elemental carbon value. The results from each apparatus were then plotted against both the Bosch number and reference elemental carbon values. The equations of the best fit lines for these plots were derived, and these gave functions for elemental carbon and Bosch number from the output of each new optical method. For each optical method, the range of DEP loadings which can be measured has been determined, and conversion equations for elemental carbon and Bosch number have been obtained. All three optical methods studied will effectively quantify blackness as a measure of elemental carbon. Of these the Magee Scientific OT21 transmissometer has the best performance. The Microcolor II and scanner/photoshop methods will in addition allow conversion to Bosch number which may be useful if historical Bosch data are available and functions for this are described. The scanner/photoshop method demonstrates a technique to obtain measurements of DEP exposure without the need to purchase specialized instrumentation.

## INTRODUCTION

Diesel exhaust particulate (DEP) has been linked with respiratory disease in workers and in 2012 IARC recognized DEP as a human carcinogen (IARC, 2012). DEP is a complex mixture of substances that can be split into two groups: organic carbon (OC) and elemental carbon (EC). OC represents all the molecular species that are either combustion products or unburned fuel constituents, for instance, benzene and polyaromatic hydrocarbons. EC is the carbon soot left over from combustion and is used as a marker for exposure to DEP (Birch and Cary, 1996; Birch and Noll, 2004, Dabill, 2005; Stewart et al., 2010; Noll et al., 2013, ). The United Kingdom does not currently have a workplace exposure limit for DEP. However, under the Control of Substances Hazardous to Health Regulations (COSHH), DEP is classed as a substance hazardous to health, and these require that exposure be prevented or, where this is not reasonably practicable, adequately controlled. Authorities in Germany have imposed a limit on exposure to DEP of 100 µg m^3^, measured as EC (TRGS 900, 1996).

As an alternative to a laboratory-based combustion method for EC in DEP, the Health and Safety Laboratory (Dabill, 2005) developed an in-field on-filter method using a Bosch meter instrument to measure reflected light to assess the darkness of a filter and therefore the amount of EC sampled. This provided the Occupational Hygienist with the potential for rapid on-site analysis of personal samples and the ability to quickly respond to high levels that may affect the health of workers. The Bosch meter is no longer manufactured, and hence, a replacement was deemed desirable. This study describes the differences between three alternative techniques to replace the Bosch meter.

Each technique uses light to measure the amount of exhaust particulate on a filter without destroying it. Two of these techniques required a dedicated instrument, the third used an office scanner to acquire an image of the filter which was then measured using the histogram function in Adobe Photoshop software (Adobe Systems Inc., San Jose, CA, USA). The dedicated instruments studied were the OT21 Transmissometer (Magee Scientific Inc., Berkeley, CA, USA), which is widely used for monitoring carbon particulates (known as black carbon) in atmospheric air (Ahmed et al., 2009; Chow et al., 2010) and a Microcolor II (DR-Lange, Dusseldorf, Germany), which is normally used for colour monitoring in manufacturing environments but also has a blackness scale similar to the Bosch meter. The electronic scanner in combination with Adobe Photoshop software technique has recently been published as an inexpensive method for monitoring carbon particulates in atmospheric air (Chang et al., 2011) and has the potential to improve the cost effectiveness and accessibility of exposure measurements for DEP.

These techniques were then compared with the Bosch meter to assess their potential as a replacement. The techniques were also directly compared with EC determined by a two-stage combustion method consistent with the European standard (EN 14530:2004) to assess ability to measure EC independently of the approach used by Dabill (2005).

## METHODS AND MATERIALS

To compare the different measurement techniques, a selection of filters with a range of DEP loadings were prepared. The analysis of EC is dependent on the DEP source (Dabill, 2005) so samples from two emission sources were collected for comparison.

Quartz fibre filters (25mm diameter QMX Whatman) were chosen as the filter substrate to ensure compatibility with the laboratory-based combustion reference method.

Filters were loaded with DEP from a gypsum mine (referred to as mine air) and the exhaust from a mobile crane (referred to as crane exhaust).

Higgins-Dewell designed cyclone samplers developed by the British Cast Iron Research Association were used to sample the mine air at a flow rate of 2.2 l min^−1^ to obtain only particulates of a diameter of 15 µm or less from the mine air; the median aerodynamic diameter sampled is 4 µm. The exhaust samples were taken from a single vessel which drew air from the exhaust outlet through a 4 m length of 6.35mm internal diameter polyvinylchloride tubing using open cowl samplers at a flow rate of 2 l min^−1^ in batches of 5 replicates. The difference in sampling methodology was a facet of the desire to collect the exhaust from the vehicles with minimum complexity as when utilizing direct sampling the use of a separation technique was unnecessary.

To establish the ability of a range of techniques to serve as alternatives to the Bosch meter method (Dabill, 2005), each loaded filter collected from the workplace was first measured using the Bosch meter. The Bosch meter illuminates the filter, then measures the reflected light, and reports a unitless number (from here on referred to as Bosch no.) from 0.0 (white) to 9.9 (black).

Three optical techniques were then evaluated as potential replacements for the Bosch meter. The results for each optical technique were then compared graphically with the readings from the Bosch meter in order to assess how suitable each would be as a replacement. The statistical significance of the differences between curves was determined using the joint Wald test.

### Instrumentation

The DR-Lange Microcolor II is an instrument designed for monitoring colour consistency in manufacturing environments. Results were obtained with a 10-mm aperture using the difference gloss measurement function on a scale of 0 (white) to−100 (black). The two colour scales (blue to yellow and red to green) were discarded.

The OT21 is well established as a technique for atmospheric air monitoring (Ahmed et al., 2009; Chow et al., 2010). The instrument measures a species described as ‘black carbon’ by absorption at a specific wavelength (880nm), and this separates it from the other optical techniques studied that measure reflected light over a range of visible wavelengths. The OT21 reports attenuation on a scale of 0 (complete transmission—white) to 500 (no transmission—black).

The use of an office scanner to measure EC has been published as a method for atmospheric air monitoring (Chang et al., 2011), and a similar idea has been used with a device attached to a smartphone camera (Ramanathan et al., 2011). The scanner used was a Toshiba e-studio 451c with Adobe Photoshop Elements 6 for processing the images. The filters were scanned and both colour and greyscale images obtained. Histograms of the stained area of the filter were generated with the Adobe software. Two histograms were available for the colour scans, RGB (red, green, and blue) and luminosity. The greyscale scans only allowed 1 histogram to be viewed. Results obtained were a unitless number between 0 (black) and 250 (white). The result was then noted manually. A chart of the results from the three histograms plotted together (Fig. 1) gave a cross comparison of the three methodologies.

**1:**
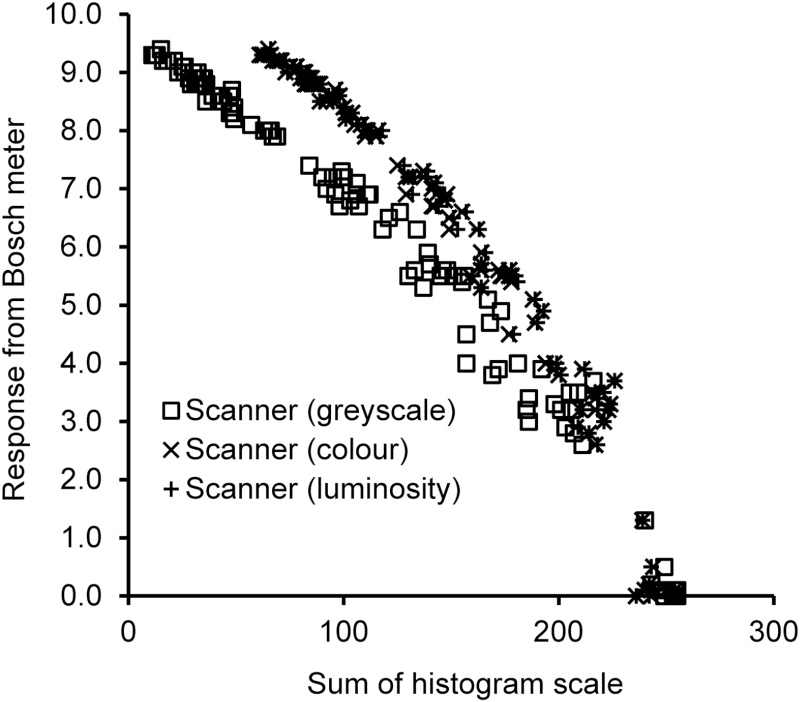
Comparison of different office scan methods with Bosch meter.

As the Bosch meter only gave a surrogate measurement of EC, because it is not directly calibrated against the analyte, a combustion method was used as a reference.

After each of the loaded filters had been measured using each technique, a combustion analysis was performed to obtain a definitive value for EC. Combustion analysis was performed following a method consistent with the EN 14530:2004 standard using an Analytik Jena multi EA3100 elemental analyser configured for carbon measurement only. Heating cycles were 700°C with gas flows of 100ml min^−1^ O_2_, 250ml min^−1^ Ar, carbon evolved classified as OC and 950°C with gas flows of 150ml min^−1^ O_2_, 200ml min^−1^ Ar, carbon evolved classified as EC. In this way, the ability of each technique to measure EC was assessed independently. We could therefore establish both how directly each technique could substitute for the Bosch meter and also how effective it was as a measure of EC.

The comparison of measurements from each technique with Bosch no. was displayed graphically and the relationship quantified from the graphs based on best fit curves. Differences between each replacement technique and the Bosch meter were then assessed by examining the variability of the values plotted. Comparisons of each technique with EC measurements from the combustion analysis were also displayed graphically. As these charts were expected to show a logarithmic relationship consistent with the Beer–Lambert law, charts of log_10_ EC against the output of the optical technique were also plotted, excluding data for EC values <1 as these produce negative log_10_ values. A log_10_ chart was not required for the OT21 measurements as the instrument takes account of the Beer–Lambert law when calculating attenuation.

## RESULTS

A total of 87 filters were obtained from the crane exhaust sampling with EC loadings ranging from 4 to 390 µg. A total of 19 filters were obtained from sampling the workplace air in a gypsum mine with EC loadings ranging from 3 to 60 µg.

### Comparisons with Bosch meter

Comparisons with the Bosch meter are shown in Figs 1 and 2. In each chart, the full range of filters are displayed, and in Fig. 2 the results are identified as belonging to one of the two sampling environments, mine air or crane exhaust.

**2:**
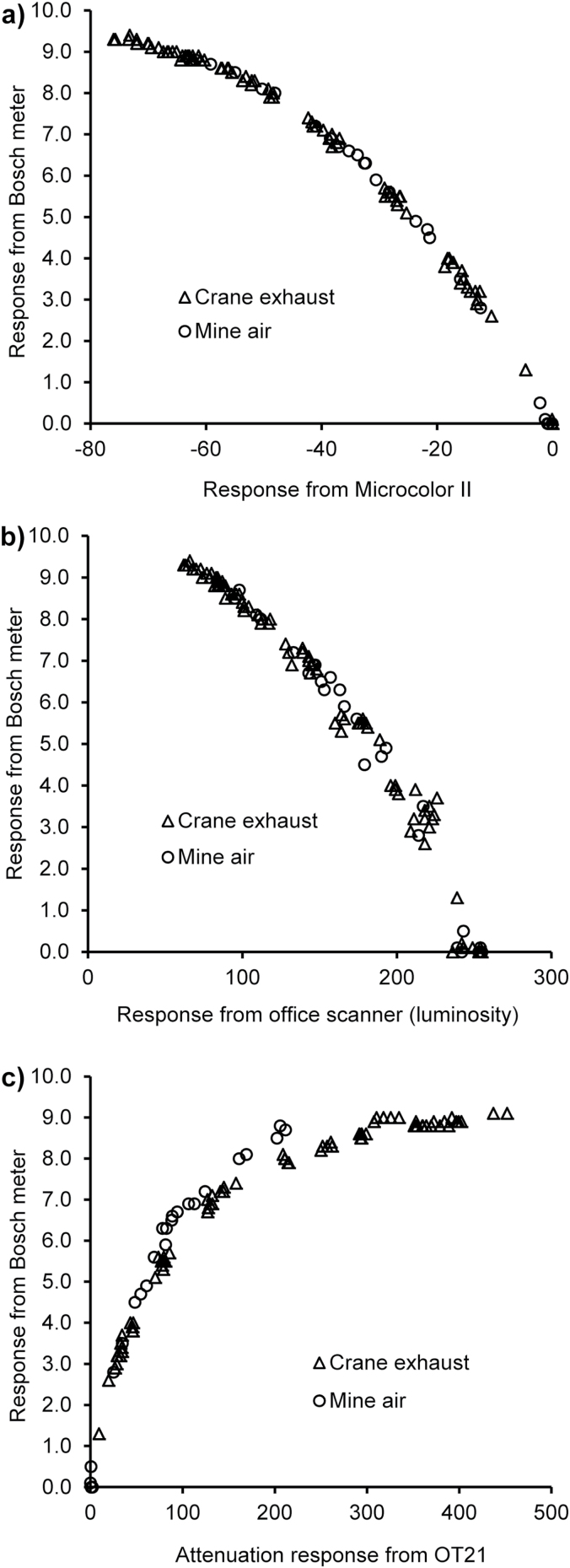
Comparisons of Bosch meter and (a) Microcolor II, (b) Scanner (luminosity), and (c) OT21.

Figure 1 shows how the three scanner methodologies compare. Examining colour and luminosity histograms of the same scan image obtains almost equivalent results (*P* = 0.364), and therefore, only luminosity results are described for other comparisons. The results from the greyscale scans differ substantially from the colour/luminosity results (*P* < 0.001), and therefore, separate equations were required to describe the relationships with Bosch no.

The charts (a) and (b) in Fig. 2 show there are good correlations between the Microcolor II and scanner (luminosity) measurements and Bosch no. For the scanner there is no statistically significant difference between the two filter sources (*P* = 0.364). In the case of the Microcolor II, statistical analysis indicates there is a significant difference between the two sources of filters (*P* < 0.001); however, it is clear from the charts and functions that the absolute magnitude of this difference is small. It can be assumed that the results from different filter sources are similar, and the significance of the difference arises from the high precision of the results and the difference in range which affects the intercept term.

In Fig. 2c, the results from the OT21 are plotted against Bosch no. Once again there is a strong correlation between the two measurements. Above a Bosch no. of 5.5, there is some indication that the two filter sources follow separate curves but the distinction is weak because there are few mine air filter results with a Bosch no. above 5.5. The nature of the relationships meant that simple functions could not be derived, and therefore, the statistical significance of the difference could not be determined.

Table 1 contains equations for the relationship each instrument has with the Bosch no. and the *R* ^2^ value quantifying how well the equation model fits the points (a coefficient of 1 = all data points perfectly match equation).

**Table 1.** Formulae for calculating Bosch no

Technique	Comparison with Bosch no.	*R* ^2^
Scanner (luminosity)	Bosch no. = −0.0002*x* ^2^ + 0.01*x* + 9.3269	0.9847
Scanner (greyscale)	Bosch no. = −9E−05*x* ^2^ − 0.0142*x* + 9.3806	0.9891
Microcolor II	Bosch no. = −0.0016*x* ^2^ − 0.2374*x* + 0.1221	0.9987
OT21	Quadratic formula not appropriate	n/a

### Comparison with EC

Figure 3 compares the instrumental response of each instrument with the EC value obtained using the European reference combustion method EN 14530:2004.

**3:**
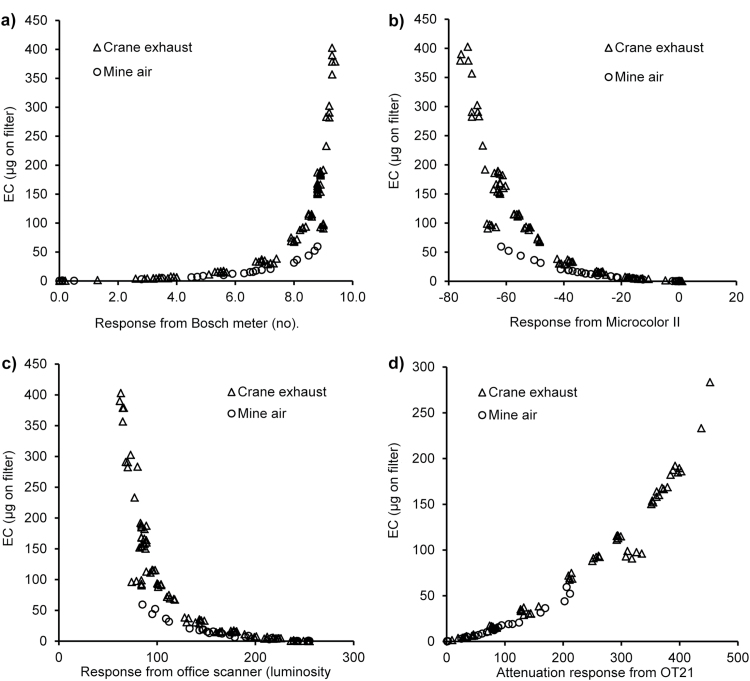
Comparison of EC and (a) Bosch meter, (b) Microcolor II, (c) Scanner (luminosity), and (d) OT21.

In Fig. 3a EC is compared with Bosch no. The chart shows a correlation; Bosch no. increases with increasing EC until a certain point where the relationship breaks down. As seen in the Bosch no./OT21 comparison, there is visual indication of separate curves for the two filter sources (*P* < 0.001).

In Fig. 3b,c the correlation with EC is also observed for the Microcolor II and scanner (luminosity) as is the divergence dependent on filter source (*P* < 0.001). The measurements from each filter source correlate better for the Microcolor II than both the scanner and the Bosch meter (see *R* ^2^ values in Tables 2, 3, and 4).

**Table 2.** Formulae for calculating EC—all filters

Technique	Best fit equation	Formula for EC	*R* ^2^	Estimated end of linearity (log_10_ EC)	Estimated maximum measurement (µg EC)
	*x* = instrument output				
Scanner (luminosity)	log_10_ EC = −0.0118*x* + 3.1563	EC = 10^(−0.118*x* + 3.1563)^	0.9459	2.0	100
Scanner (greyscale)	log_10_ EC = −0.0093*x* + 2.4524	EC = 10^(−0.0093*x* + 2.4524)^	0.9438	2.0	100
Microcolor II	log_10_ EC = −0.0308*x* + 0.239	EC = 10^(−0.0308*x* + 0.239)^	0.9596	Not determined	150
Bosch	log_10_ EC = 0.2305*x* − 0.1193	EC = 10^(0.2305*x* − 0.1193)^	0.9301	1.8	63
OT21	EC = 0.001*x* ^2^ + 0.0749*x*		0.9786	n/a	240

OT21 attenuation measurements are compared with EC in Fig. 3d. A correlation with EC is again observed; there is a difference between the two filter sources (*P* < 0.001), but this is of a lower magnitude than that observed for the other methods.

The chart of EC plotted against measurements from each technique (except the OT21) are exponential, and this is as expected because each relies on the measurement of reflected light and as loadings increase measurements will approach a point where no light is reflected, but loadings can still be increased past this point and so EC can continue to increase with no increase in the optical measurement. The OT21 is different in that it measures the light absorbed by the sample rather than reflected. The exponential relationship still applies, but it is hidden in the measurements as the attenuation result is a natural logarithm of the relative intensity after absorption.

The log_10_ value for each of the EC results was calculated and plotted against the measurements from the Bosch meter, Microcolor II, and scanner (luminosity) to give linear charts. In this way an equation to calculate EC from the optical result could be derived and an estimate of the limit of the useful range made from the point at which the data deviates from linearity. The deviation from linearity was calculated for the Bosch meter and scanner by fitting a restricted cubic spline to the data and then calculating the change in slope, this is constant for much of the data before increasing abruptly, therefore the point when the change in slope increases was taken as the point at which the data deviates from linearity. This method was not appropriate for the Microcolor II and OT21. The log_10_ chart for the Microcolor II is linear throughout the data set, and the OT21 versus EC chart (Fig. 3d) incorporates the logarithm and is non-linear. In these cases, the end of the useful range was estimated by examination of the EC charts (Fig. 3c,d) from the region of the chart where the change in EC becomes large compared with the change in instrument output. Equations and *R* ^2^ values for the scanner and Microcolor II are calculated from all the data points, whereas only the linear section of the data was used for the Bosch calculation. The Bosch calculations are therefore based on fewer data points, and the *R* ^2^ values in the tables are artificially high. Table 2 shows the equations and *R* ^2^ values derived for calculating EC from the measurements from each of the techniques. Tables 3 and 4 show the equations and *R* ^2^ values as individual data sets for the cyclone filters and crane filters, respectively, and these functions were used to calculate the statistical significance of the differences described above. The *R* ^2^ values increase significantly when the data sets are isolated based on filter source. In the crane filter data set, the *R* ^2^ values are further increased if the outlying data points at ~100 µg EC are disregarded as shown in Table 4 (not shown for the Bosch meter as 100 µg is outside the linear range).

**Table 3.** Formulae for calculating EC—mine air filters

Technique	Best fit equation	Formula for EC	*R* ^2^	Estimated end of linearity (log_10_ EC)	Estimated maximum measurement (µg EC)
	x = instrument output				
Scanner (luminosity)	log_10_ EC = −0.0087*x* + 2.5126	EC = 10^(−0.0087*x* + 2.5126)^	0.9578	2.0	100
Scanner (greyscale)	log_10_ EC = −0.0065*x* + 1.9672	EC = 10^(−0.0065*x* + 1.9672)^	0.9596	2.0	100
Microcolor II	log_10_ EC = −0.0244*x* + 0.3196	EC = 10^(−0.0244*x* + 0.3196)^	0.9852	Not determined	150
Bosch	log_10_ EC = 0.2*x* − 0.0795	EC = 10^(0.2*x* − 0.0795)^	0.9805	1.8	63
OT21	EC = 0.0007*x* ^2^ + 0.098*x*		0.9771	n/a	n/a

**Table 4.** Formulae for calculating EC—crane exhaust filters

Technique	Best fit equation	Formula for EC	*R* ^2^		Estimated end of linearity (log_10_ EC)	Estimated maximum measurement (µg EC)
	x = instrument output		All results	Without outliers		
Scanner (luminosity)	log_10_ EC = −0.0118*x* + 3.1992	EC = 10^(−0.118*x* + 3.1992)^	0.9710	0.9830	2.0	100
Scanner (greyscale)	log_10_ EC = −0.0093*x* + 2.4973	EC = 10^(−0.0093*x* + 2.4973)^	0.9657	0.9757	2.0	100
Microcolor II	log_10_ EC = −0.0306*x* + 0.2837	EC = 10^(−0.0306*x* + 0.2837)^	0.9743	0.9907	Not determined	150
Bosch	log_10_ EC = 0.244*x* − 0.1399	EC = 10^(0.244*x* − 0.1399)^	0.9823	n/a	1.8	63
OT21	EC = 0.0011*x* ^2^ + 0.0348*x* + 4.9405		0.9765	0.9910	n/a	240

## DISCUSSION

The term ‘filter source’ is used to describe all facets of the filter sample, including any interferences, sampling artefacts, deposition effects, and DEP source characteristics. For this reason, this work does not propose that the results show a source dependency for the DEP measured, only the filters measured.

### Comparisons with Bosch meter

The study investigates the suitability of replacements for the Bosch meter. Each of the three optical techniques has a strong correlation with Bosch no. None is a direct 1:1 replacement as the charts are non-linear, and therefore, an equation for a conversion function is required. In the scanner and Microcolor II techniques, the correlation is independent of the sources of filters loaded with DEP as shown in Fig. 2a,b.

All the techniques studied could provide a replacement for the Bosch meter for indicative field measurements to provide a fast response to control exposure as the EC content could be inferred from each of them. The equations derived and shown in Table 1 for the relationships between Bosch no. and each of the techniques allows the calculation of Bosch no. from the scanner and Microcolor II results. This would be useful where an existing Bosch no. to EC calibration is available. Although the curves defining the relationships are quite different for the greyscale and colour/luminosity scans, the coefficients of determination are very similar (Table 1), indicating that each would serve equally well as a Bosch meter replacement. The Microcolor II has the best relationship with Bosch no. as shown by the *R* ^2^ value.

In this work, the *R* ^2^ values in Table 1 show that the Microcolor II (0.9987) is a more repeatable technique than the scanner (0.9847); the results for two filters with the same EC loading are likely to be more similar to each other; and this is also displayed graphically in the spread of data points in the charts. The curved nature of the relationships with Bosch no. also serve to demonstrate the increased precision of the replacement techniques at higher loadings because the increasing curve shows that the replacement techniques are still giving different measurements when the Bosch meter has reached the top of its range. In the Microcolor II chart, we can see that in the first half of the instruments range Bosch no.’s 0–7 are covered and the second half describes 7–9.5.

It is possible that the OT21 measurements may show some dependency on filter source in its relationship with Bosch no. From Fig. 3c, it is observed that the above an attenuation value of 100 the results from the two filter sources may follow slightly different curves although it was not possible to determine the statistical significance of the difference because simple functions for the curves could not be derived. This increased specificity is consistent with the OT21 measuring at a single wavelength of 880nm (Hansen et al., 1984) identified as specific to black carbon. Calculating the standardized residuals showed that a quadratic function as used to calculate Bosch no. from the other techniques did not fit the data from the OT21. A fractional polynomial (degree 3) function fitted the data well but is overly complex for routine use. The demonstration that a quadratic function is not appropriate for comparison of the OT21 with Bosch no. when it is appropriate for the scanner and Microcolor II combined with the specificity of the measurement wavelength supports the implication that it is a fundamentally separate technique. For these reasons, no conversion equation is given in Table 1, and the OT21 is considered by the author unsuitable as a like for like replacement for the Bosch meter but is a useful alternative as it does measure EC using a different methodology.

### Elemental carbon

The Bosch meter was established as a convenient method of monitoring DEP without the delay and cost associated with laboratory analysis. This was predicated on the relationship between Bosch no. and EC, which is the European standard method for measuring DEP (EN 14530:2004). Preparing charts and relationships of each of the techniques against EC allowed a comparison with the Bosch meter and reference method measuring EC.

In this comparison, the weaknesses of the Bosch method, and all the optical methods which emulate it, are apparent. Filter source has an effect on the relationship with EC for all the methods. The effect is greater at the higher end of the EC loading range. For example, a mine air filter has a Bosch no. of 8.1 and an EC loading of 36; a crane exhaust filter with a Bosch no. of 8.0 has an EC loading of 72. This is important as normally it is expected that measurement uncertainty declines with higher measurement values, and it also further deteriorates the performance of those techniques with an already narrow measurement range.

The dependency on filter source observed in the relationship with EC for Bosch no., scanner, and Microcolor II is consistent with that found for Bosch no. by Dabill (2005). In this case, however, the differences observed may arise from the different samplers used rather than directly from the source. The cyclones deposit more dust in the centre of the filter than the open faced samplers; any analysis method which does not view the whole filter may give greater sensitivity for the mine air results (that were obtained using a cyclone sampler). This is supported by the evidence that the variation in results only becomes apparent at higher loadings where the contrast between the density of deposit is greater. Nevertheless it is advantageous if a method can be shown to be independent of this effect. It is possible that respirable mine dust was collected on the filters, and this could have been a source of interference in the optical measurements. The addition of a submicron size selector (developed by the US Bureau of Mines and optimized by NIOSH) was considered but discounted on the grounds of cost and with the intention of developing a method which would not require it.

The OT21 shows the most consistency between filter sources, and this is unsurprising when it is understood that it is a specific technique and therefore unlike the other optical methods and that it also has the worst relationship with the Bosch meter.

Having taken logarithms of the EC values the charts of these plotted against the measurements from each technique allowed the establishment of functions to calculate EC from a measurement by any of the optical techniques.

Because there was a dependency on filter source, these functions were calculated for all filters and also for the mine air and crane exhaust filters individually. The coefficients of determination and changes in gradient between all filters and individual sources show that there is a definite dependency on filter source for the EC relationship. This is less clear for the OT21 where the crane only equation is similar to the all filters equation. This may however be a result of the increased measurement range of the OT21 and the higher loadings of the crane filters having a determining effect on the all filters curve as the function for the cyclone only curve is slightly different. A divergence might still be observed if higher loading cyclone filters were available. The OT21 showing greater consistency between sources in the EC relationships is consistent with the poor performance in the comparison with the Bosch meter because the Bosch is poor at measuring EC from different sources. The range of analysis improves from the Bosch meter through the scanner and Microcolor II to the OT21. Whether the limitations in analytical range represent a problem for the determination of EC on filters from real workplace environments is dependent on what concentrations of EC exist in those environments combined with the sampling protocols used. As an example, taking the 100 µg m^−3^ (TRGS 900) guidance value used in Germany, a typical 4-h, half-shift sampling period at 2.2 l min^−1^ using the cyclone sampler will produce a sampled air volume of 2.2×240 = 528 l = 0.528 m^3^ and a filter loading of 100×0.528 = 53 µg. This is within the range of all the techniques and, as such, the analytical range would not normally be the primary criterion when selecting a technique.

## CONCLUSION

All the techniques investigated provide practical options for the measurement of DEP as EC and therefore as replacements for the Bosch meter.

### Substituting for the Bosch meter

The Microcolor II instrument has the best correlation with the Bosch no. and is therefore the best like for like replacement. Although this particular instrument is no longer manufactured, the results demonstrate the effectiveness of the difference gloss measurement technique and other instruments utilizing this technique are available.

The scanner technique is less precise and repeatable but nevertheless is suitable as a general monitoring technique. Because there is no need to purchase a dedicated instrument, this technique allows access to DEP measurements for non-traditional users.

The data comparing Bosch no. with the output from the scanner show that Bosch no. can be calculated from the scanner or Microcolor II result without the source of the DEP having an effect. It is therefore practicable for a site that has a calibration for EC from Bosch no. to move to either technique as a replacement and calculate EC by calculating Bosch no. from scanned luminosity or Microcolor II output first. However, this extra step adds a degree of uncertainty that could affect the accuracy of results. Because the analytical technique is non-destructive, best practice would be to use calculation of Bosch no. as an interim procedure whilst a set of filters is gradually built up that can then be analysed by combustion to prepare a direct scanned luminosity or Microcolor II measurement to EC calibration.

The OT21 uses a different measurement technology and as such is unsuitable as a like for like substitute for the Bosch meter but can be used as an alternative.

### Measuring EC directly

The three reflected light techniques, scanner, Bosch meter, and Microcolor II, have comparable performance when measuring EC, the transmitted light technique, the OT21, has improved performance. The results from each require calibration for each filter source. Of the three reflected light techniques, the Microcolor II is more reproducible (the higher *R* ^2^ means that each result is closer to the calibration function). The OT21 is a significant improvement on the three other optical techniques for measuring EC; the *R* ^2^ is significantly higher, especially if the five outlier results are discounted, and the range of up to 240 µg EC represents a significant advantage over the three reflected light techniques.

The DR-Lange and Magee instruments have a higher limit at which they can accurately measure EC than the scanner technique.

If precision and range are important factors, then the DR-Lange Microcolor II instrument and the Magee OT21 Transmissometer should be considered the most appropriate instruments. For general monitoring, the scanner technique is acceptable. All techniques require calibration against EC for each site and sampling method, but if a site has a Bosch no. to EC calibration, EC may be calculated as described above. There is evidence to suggest that the requirement for site-specific calibration with EC may be relaxed for the OT21 if the associated increased uncertainty in the measurement can be accepted.

The DR-Lange Microcolor II and the Magee OT21 Transmissometer each cost in the region of £8000, whereas the scanner technique uses only standard office hardware and a commonly available software program. The scanner therefore represents a method for monitoring DEP in workplaces in a more convenient way using equipment that is readily available and so could potentially broaden the range of workplace monitoring.

## FUNDING

Health and Safety Executive. Its contents, including any opinions and/or conclusions expressed, are those of the author alone and do not necessarily reflect HSE policy.
